# Psychiatric and non-psychiatric polypharmacy among older adults with schizophrenia: Trends from a population-based study between 2000 and 2016

**DOI:** 10.3389/fphar.2023.1080073

**Published:** 2023-02-07

**Authors:** Carlotta Lunghi, Louis Rochette, Victoria Massamba, Isabelle Tardif, Amina Ouali, Caroline Sirois

**Affiliations:** ^1^ Department of Health Sciences, Université du Québec à Rimouski, Lévis, QC, Canada; ^2^ Population Health and Optimal Health Practices, CHU de Québec - Université Laval Research Center, Québec, QC, Canada; ^3^ Department of Medical and Surgical Sciences, University of Bologna, Bologna, Italy; ^4^ Institut national de Santé Publique du Québec, Québec, QC, Canada; ^5^ Faculty of Medicine, Université Laval, Québec, QC, Canada; ^6^ Faculty of Pharmacy, Université Laval, Québec, QC, Canada; ^7^ Quebec Excellence Centre on Aging, VITAM Research Centre on Sustainable Health, Québec, QC, Canada

**Keywords:** polypharmacy, drug utilization, administrative databases, trends, older adults, elderly, schizophrenia

## Abstract

**Background:** Schizophrenia is a severe psychiatric disorder associated with multiple psychiatric and non-psychiatric comorbidities. As adults with schizophrenia age, they may use many medications, i.e., have polypharmacy. While psychiatric polypharmacy is well documented, little is known about trends and patterns of global polypharmacy. This study aimed to draw a portrait of polypharmacy among older adults with schizophrenia from 2000 to 2016.

**Methods:** This population-based cohort study was conducted using the data of the Quebec Integrated Chronic Disease Surveillance System of the National Institute of Public Health of Quebec to characterize recent trends and patterns of medication use according to age and sex. We identified all Quebec residents over 65 years with an ICD-9 or ICD-10 diagnosis of schizophrenia between 2000 and 2016. We calculated the total number of medications used by every individual each year and the age-standardized proportion of individuals with polypharmacy, as defined by the usage of 5+, 10+, 15+, and 20+ different medications yearly. We identified the clinical and socio-demographic factors associated with polypharmacy using robust Poisson regression models considering the correlation of the responses between subjects and analyzed trends in the prevalence of different degrees of polypharmacy.

**Results:** From 2000 to 2016, the median number of medications consumed yearly rose from 8 in 2000 to 11 in 2016. The age-standardized proportion of people exposed to different degrees of polypharmacy also increased from 2000 to 2016: 5+ drugs: 76.6%–89.3%; 10+ drugs: 36.9%–62.2%; 15+: 13.3%–34.4%; 20+: 3.9%–14.4%. Non-antipsychotic drugs essentially drove the rise in polypharmacy since the number of antipsychotics remained stable (mean number of antipsychotics consumed: 1.51 in 2000 vs. 1.67 in 2016). In the multivariate regression, one of the main clinically significant factor associated with polypharmacy was the number of comorbidities (e.g., Polypharmacy-10+: RR_[2 VS. 0–1]_ = 1.4; 99% IC:1.3–1.4, RR_[3–4]_ = 1.7 (1.7–1.8); RR_[5+]_ = 2.1 (2.1–2.2); Polypharmacy-15+: RR_[2 VS 0–1]_ = 1.6; 99% IC:1.5–1.7, RR_[3–4]_ = 2.5 (2.3–2.7); RR_[5+]_ = 4.1 (3.8–4.5).

**Conclusion:** There was a noticeable increase in polypharmacy exposure among older adults with schizophrenia in recent years, mainly driven by non-antipsychotic medications. This raises concerns about the growing risks for adverse effects and drug-drug interactions in this vulnerable population.

## Introduction

Schizophrenia is a severe disease characterized by hallucinations, delusions, disorganized speech, and abnormal thinking, which significantly impact the ability of patients to function in their daily lives and quality of life (Marder and Cannon, 2019). It is among the top 10 global causes of disability (Fleischhacker et al., 2014; Charlson et al., 2018), with an estimated worldwide prevalence that can reach up to 1% (Saha et al., 2005). Schizophrenia patients are more often sedentary, higher cigarette smokers and drug users (Fleischhacker et al., 2014). They have frequent physical comorbidities such as cardiovascular diseases (Fleischhacker et al., 2014), obesity (Mamakou et al., 2018), type two diabetes (Fleischhacker et al., 2014), metabolic syndrome (Jeon and Kim, 2017), and dementia (Stroup et al., 2021). Mental comorbidities such as depression (Remington et al., 2017), alcohol or substance abuse (Buchanan et al., 2010), and insomnia (Stummer et al., 2018) are also common in these patients. It is also hypothesized that the aging process is accelerated in schizophrenia patients (Nguyen et al., 2018).

On the other hand, patients with schizophrenia are underdiagnosed with physical conditions and, when the diagnosis arrives, these conditions are often at an advanced stage, requiring more intensive treatment and more medications (Fleischhacker et al., 2014). In the general older population, multimorbidity (Gontijo Guerra et al., 2019), is often associated with polypharmacy [i.e., taking multiple medications (Sirois et al., 2019a)]. Polypharmacy is a genuine concern in older individuals due to the higher risk for adverse drug events, drug-drug interactions, adherence problems, and potentially inappropriate prescriptions (Kojima et al., 2020; Lin, 2020). In recent years, polypharmacy has been studied in different populations of older individuals with chronic conditions, such as heart failure (Campeau Calfat et al., 2022), chronic obstructive pulmonary disease (COPD) (Sirois et al., 2019b), or diabetes (Oktora et al., 2021). These studies have shown an increase in polypharmacy in the last decades. Nevertheless, polypharmacy has not been studied in older patients with schizophrenia, despite this concern similarly exists for these patients because of their elevated risk of multimorbidity (Buchanan et al., 2010; Fleischhacker et al., 2014; Jeon and Kim, 2017; Remington et al., 2017; Mamakou et al., 2018; Nguyen et al., 2018; Stummer et al., 2018; Stroup et al., 2021).

The cornerstone of schizophrenia treatment is antipsychotic medications (Marder and Cannon, 2019). Antipsychotic drugs usually must be taken lifelong (Remington et al., 2017; Marder and Cannon, 2019). Antipsychotic polypharmacy (Jeon and Kim, 2017), namely the use of more than one antipsychotic at the same time, is frequent in clinical practice either to achieve reasonable control of psychosis or to treat specific symptoms such as insomnia (Stummer et al., 2018) or other side effects (Baandrup, 2020). In a recent study on hospitalized patients with schizophrenia-spectrum disorders, 28.1% of patients took four or more psychotropic drugs before hospitalization, with a mean number of 2.8 medications. Still, the number of non-psychotropic drugs was not mentioned (Gaudiano et al., 2018). We can hypothesize that global polypharmacy is significant, especially in older patients with schizophrenia, given multimorbidity, as age is a predictor of polypharmacy in psychiatric patients (Viola et al., 2004; Paudel et al., 2020).

Even if global polypharmacy may be frequent in older patients with schizophrenia, studies on this topic have focused only on the psychiatric polypharmacy (Zink et al., 2010), with the main emphasis on the antipsychotic combination therapy (Gaudiano et al., 2018; Baandrup, 2020; Lin, 2020). Considering the potential burden that polypharmacy may impose on these patients, it is important to quantify the problem and to identify factors associated with polypharmacy that may help identify those at higher risk of adverse consequences of polypharmacy. To the best of our knowledge, no study has investigated the trends and patterns of global polypharmacy in older adults with schizophrenia.

The objectives of this study were thus to draw a portrait of polypharmacy among Quebec older adults with schizophrenia from 2000 to 2016 and to identify factors associated with different degrees of polypharmacy.

## Materials and methods

### Data source and population

We performed a population-based observational study of annual cohorts (one cohort for each year under study) using the data of the Quebec Integrated Chronic Disease Surveillance System (QICDSS) of the National Institute of Public Health of Quebec (*Institut National de Santé Publique du Québec*−INSPQ) (Blais et al., 2014). The QICDSS database is composed of five different sources of medico-administrative data: information on the insurance plan of its members (i.e., starting and end date of eligibility), on hospitalizations (i.e., primary and secondary diagnostic codes according to the ninth and tenth revisions of the International classification of diseases–ICD-9 and ICD-10, respectively), on physician visits (primary ICD-9 diagnostic codes), on reimbursed drugs (i.e., drug name, dispensing date, days’ supply, the specialty of the prescriber) and on deaths. More than 90% of the Quebec population aged 65 years and above is covered by the public drug plan, and their information is in the QICDSS (Blais et al., 2014). Older adults in long-term care and those with a private drug plan are not covered by the public drug plan and are thus excluded.

This study identified all Quebec residents over 65 with an ICD-9 or ICD-10 diagnostic inpatient or outpatient code for schizophrenia (ICD-9: 295.0 to 295.9; ICD-10: F20.0 to F21.9, F23.2, F25.0 to F25.9) between April 1st, 2000 and March 31st, 2017. We constructed 17 cohorts (one for each year under study) which included both incident and prevalent cases of schizophrenia.

### Definition of polypharmacy and medication use

We assessed the number of different medications used by each individual in every fiscal year, with the fiscal year beginning on April 1st and ending on March 31st. We included all the patients covered by the public drug insurance plan and alive for the year under investigation to assess the total number of medications used that year. Medications were classified according to the American Hospital Formulary Service (AHFS) classification (Francke, 1963) and common drug denomination (chemical name of the medication).

There is no consensus on the definition of polypharmacy, with the most common definitions used in the literature having a threshold of 5 or 10 medications (Sirois et al., 2019a). Considering the population of older adults with multimorbidity, we decided to use different thresholds to define polypharmacy. Thus, in this study, polypharmacy was referred to as the presence of prescription claims for at least 5, 10, 15, or 20 different medications in a fiscal year. We, therefore, assessed different degrees of polypharmacy for the time frame of a fiscal year, for every fiscal year in the study period. In accounting for the sum of medications claimed in each fiscal year, we considered only medications reimbursed by the public drug plan. Thus, over-the-counter drugs or other non-reimbursed medications (e.g., z-drugs) were not included. Medications used as needed (“prn”) and those for acute illnesses (e.g., antibiotics), if reimbursed by the public drug plan, were included.

### Socio-demographic and clinical characteristics

Socio-demographic characteristics of individuals in each cohort included age, sex, material and social deprivation index (in quintiles) and residence area [based on the Quebec census geographical areas: Montreal (> 1,000,000 inhabitants), other census metropolitan (100,000 to 1,000,000 inhabitants), agglomerations (10,000 to 100,000 inhabitants), and rural (< 10,000 inhabitants)]. Material and social deprivation indexes represent a proxy of the socioeconomic status of the individual (Pampalon et al., 2009). These indexes, which are ecological indexes based on the census dissemination area, are divided into quintiles, with the first quintile including the least deprived and the fifth quintile the most deprived individuals (Pampalon et al., 2009). We also calculated a global deprivation index combining social and material deprivations according to five classes (most deprived, deprived, mostly socially deprived, mostly materially deprived, least deprived), as explained in Supplementary Figure S1. We identified the annual number of hospitalizations and the number of physician visits recorded in the QIDSS for each individual and each year under study. ICD-9 and ICD-10 codes were used to identify comorbid conditions according to validated QICDSS algorithms for Alzheimer’s disease, asthma, chronic obstructive pulmonary disease (COPD), diabetes, hypertension, mood disorders, osteoporosis, stroke, mood disorders, and dementia (Blais et al., 2014) during 5 years (the current year and the four preceding years). We used the combined Charlson-Elixhauser comorbidity index to calculate a score of the burden of comorbidities of each patient (Simard et al., 2018).

### Statistical analysis

We used descriptive statistics to describe the subjects included in each cohort. For each subject, we assessed the number of different drugs claimed in every fiscal year by using the drug’s common denomination (identifying the chemical entity). We calculated the proportion of individuals exposed to different degrees of polypharmacy and then estimated the age-standardized annual prevalence of polypharmacy with the reference population of Quebec in 2011. We further identified the clinical and socio-demographic factors associated with polypharmacy using robust Poisson regression analyses, modeling the number of individuals who claimed at least 5, 10, 15, or 20 different medications in a fiscal year, depending on the model and considering the correlation of the responses between subjects. Thus, we calculated unadjusted and adjusted prevalence ratios (PRs) and their 99% confidence intervals (CIs). We also tested the trends of change in the mean annual prevalence of polypharmacy with the same models. We performed all the analyses using SAS Enterprise Guide 7.1.

## Results

Cohorts comprised 2,566 individuals in 2000 and up to 4,634 in 2016 (Table 1). Female patients were the large majority (about two-third of each cohort), as for those in the 66–75 age group (about 70%), with some changes in the age distribution depending on the cohort. Indeed, the proportion of older individuals (>85 years) slightly increased from 4.2% to 5.9% over time. Between 2000 and 2016, the proportion of individuals with mood disorders decreased by 26% (from 40.3% to 30.0%), but those with diabetes, hypertension and osteoporosis largely increased, with relative changes of 104%, 54%, and 124%, respectively. Indeed, as expected, with the aging of the individuals being part of the annual cohorts from 2000 to 2016, the population was composed of older individuals with more physical comorbidities in more recent years.

**TABLE 1:** Characteristics of the population studied from selected cohorts (2000; 2004; 2008; 2012 and 2016).

Characteristics	2000		2004		2008		2012		2016	
	*n* = 2,566		*n* = 2,947		*n* = 3,467		*n* = 4,100		*n* = 4,634	
	N	%	N	%	N	%	N	%	N	%
Age (years)										
66–75	1,832	71.4	2,037	69.1	2,326	67.1	2,849	69.5	3,283	70.9
76–85	627	24.4	773	26.2	960	27.7	1,020	24.9	1,079	23.3
86+	107	4.2	137	4.7	181	5.2	231	5.6	272	5.9
Sex										
Female	1,780	69.4	1,971	66.9	2,284	65.9	2,652	64.7	2,846	61.4
Male	786	30.6	976	33.1	1,183	34.1	1,448	35.3	1,788	38.6
Material deprivation (quintile)										
1 (least deprived)	380	14.8	347	11.8	458	13.2	524	12.8	586	12.7
2	407	15.9	433	14.7	530	15.3	562	13.7	628	13.6
3	455	17.7	526	17.9	619	17.9	628	15.3	721	15.6
4	448	17.5	588	20.0	670	19.3	794	19.4	868	18.7
5 (most deprived)	585	22.8	620	21.0	740	21.3	879	21.4	1,012	21.8
Missing	291	11.3	433	14.7	450	13.0	713	17.4	819	17.7
Social deprivation (quintile)										
1 (least deprived)	275	10.7	260	8.8	328	9.5	372	9.1	425	9.2
2	309	12.0	359	12.2	399	11.5	422	10.3	513	11.1
3	407	15.9	448	15.2	527	15.2	575	14.0	574	12.4
4	521	20.3	590	20.0	715	20.6	874	21.3	998	21.5
5 (most deprived)	763	29.7	857	29.1	1,048	30.2	1,144	27.9	1,305	28.2
Missing	291	11.3	433	14.7	450	13.0	713	17.4	819	17.7
Comorbidity										
Alzheimer	247	9.6	333	11.3	495	14.28	629	15.34	606	13.1
Asthma	144	5.6	223	7.6	332	9.58	424	10.34	499	10.8
COPD	533	20.8	725	24.6	901	25.99	1,107	27.00	1,348	29.1
Diabetes	405	15.8	612	20.8	883	25.5	1,175	28.7	1,492	32.2
Heart Failure	191	7.4	234	7.9	270	7.8	345	8.4	389	8.4
Hypertension	946	36.9	1,388	47.1	1799	51.9	2,316	56.5	2,638	56.9
Mood disorders	1,034	40.3	1,150	39.0	1,352	39.0	1,372	33.5	1,390	30.0
Osteoporosis	305	11.9	508	17.2	777	22.4	1,098	26.8	1,235	26.7
Stroke	171	6.7	238	8.1	323	9.3	350	8.5	404	8.7
Number of comorbidities^a^										
0–1	615	24.0	688	23.4	761	22.0	923	22.5	1,143	24.7
2	623	24.3	650	22.1	773	22.3	893	21.8	901	19.4
3–4	779	30.4	907	30.8	1,045	30.1	1,052	25.7	1,223	26.4
5+	549	21.4	702	23.8	888	25.6	1,232	30.1	1,367	29.5
Combined comorbidity score^b^										
0	183	7.1	206	7.0	1,607	46.4	1,848	45.1	2,106	45.5
1	1,144	44.6	1,294	43.9	424	12.2	464	11.3	495	10.7
2	324	12.6	360	12.2	434	12.5	491	12.0	505	10.9
3+	915	35.7	1,087	36.9	1,002	28.9	1,297	31.6	1,528	33.0
Number of hospitalizations^c^										
0	1,567	61.1	1,862	63.2	2,233	64.4	2,660	64.9	3,055	65.9
1	642	25.0	684	23.2	774	22.3	899	21.9	984	21.2
2+	357	13.9	401	13.6	460	13.3	541	13.2	595	12.8
Number of physician visits^c^										
0	227	8.9	289	9.8	266	7.7	276	6.7	318	6.9
1–4	742	28.9	989	33.6	1,327	38.3	1,677	40.9	2027	43.7
5–9	629	24.5	702	23.8	849	24.5	1,023	25.0	1,088	23.5
10+	968	37.7	967	32.8	1,025	29.6	1,124	27.4	1,201	25.9

^a^ Number of physical and psychiatric comorbidities in a 5-years period (the current year and the 4-years before).

^b^ Charlson—Elixhauer combined comorbidity score measured in a 5-years period (the current year and the 4-years before).

^c^ Number of physician visits and hospitalizations in the current year.

**FIGURE 1:**
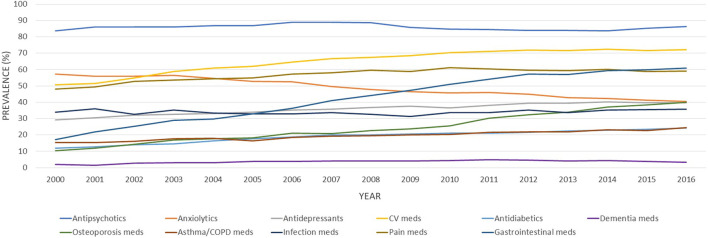
Age-standardized proportions of older adults with schizophrenia exposed to different degrees of polypharmacy (≥5, ≥10, ≥15, and ≥20 medications), between 2000 and 2016. Bars represent the age-adjusted prevalence of different degrees of polypharmacy and the line is the mean number of different medications claimed in the current fiscal year.

As reported in Figure 1, the number of different medications claimed increased over the 16 years, with the mean number of drugs claimed rising from 8.76 [standard deviation (SD) 5.29] in 2000 to 12.3 (SD 6.78) in 2016. Accordingly, the age-standardized prevalence of different degrees of polypharmacy also increased over time, with 36.9% of individuals being exposed to 10 drugs and above in 2000, increasing to 62.2% in 2016. Similarly, the prevalence of polypharmacy defined as 5 medications and above, as 15 medications and above, and as 20 medications and above went from 76.6%, 13.3%, and 3.9% in 2000 to 89.3%, 34.4%, and 14.4% in 2016, respectively (Figure 1). The trend analyses showed that the age-adjusted proportion of individuals exposed to different degrees of polypharmacy increased in the study period. Over the 17 years under investigation, the yearly mean increases of individuals exposed to varying degrees of polypharmacy were 0.8% (99% CI = 0.7%–0.9%) for 5 and more medications, 2.6% (99% CI = 2.4%–2.9%) for 10 medications and above, 4.5% (99% CI = 4.0%–4.9%) for 15 medications and above, and 5.2% (99% CI = 4.4%–5.9%) for 20 medications and above.

**FIGURE 2:**
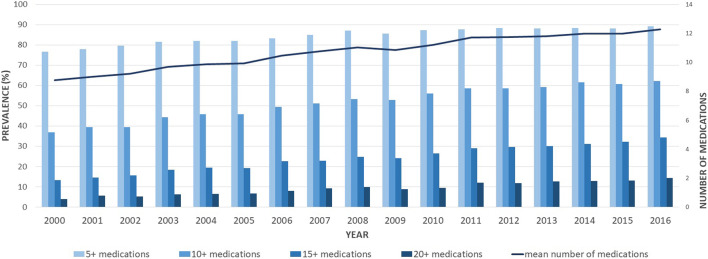
Age-standardized proportions of older adults with schizophrenia exposed to different classes of medications between 2000 and 2016. CV, cardiovascular; COPD, chronic obstructive pulmonary disease; Meds, medications.

The rise in medication use was essentially driven by non-antipsychotic drugs, as presented in Table 2. The number of antipsychotics remained stable, with a mean number of antipsychotics consumed of 1.51 ± 0.75 in 2000 and 1.67 (±0.84) in 2016. When analyzing the prevalence of the main medication classes claimed, different patterns emerged. Some classes increased over the study period, such as cardiovascular medications, gastrointestinal medications (mainly driven by proton pump inhibitors–PPIs), and osteoporosis medications (Figure 2). Other classes, such as anxiolytics, showed an important decrease overtime. The more impacting diseases were cardiovascular and respiratory comorbidities, such as heart failure, stroke, asthma, and COPD.

**TABLE 2:** Number of different antipsychotic medications claimed during one year-period by older people with schizophrenia from 2000 to 2016.

Number of antipsychotic medications	2000		2004		2008		2012		2016	
	*n* = 2,566		*n* = 2,947		*n* = 3,467		*n* = 4,100		*n* = 4,634	
	N	%	N	%	N	%	N	%	N	%
Mean ± SD	1.51 ± 0.75		1.52 ± 0.74		1.54 ± 0.75		1.62 ± 0.77		1.67 ± 0.84	
0	419	16.3	387	13.1	398	11.5	664	16.2	642	13.9
1	1,321	51.5	1,552	52.7	1,791	51.7	1,836	44.8	2,060	44.5
2	607	23.7	748	25.4	964	27.8	1,168	28.5	1,360	29.4
3+	219	8.5	260	8.8	314	9.1	432	10.5	572	12.3

SD: standard deviation

In the multivariable robust Poisson regressions, women were more likely to be exposed to polypharmacy with adjusted prevalence ratios (PR) ranging from 1.05 for 5 to 1.22 for 20 medications and above (Table 3). Older individuals were slightly more likely to be exposed to lower degrees of polypharmacy (5+ and 10+ medications), while age was not a statistically significant factor for higher levels of polypharmacy (15+ and 20+). The only clinically significant factor statistically associated with polypharmacy was the number of comorbidities, with prevalence ratios increasing with the number of comorbidities and the degree of polypharmacy (see Table 3).

**TABLE 3:** Multivariable robust Poisson regressions of the factors associated with polypharmacy, defined as the claim of at least five, ten, fifteen, or twenty different medications in 1 year during the study period.

	Polypharmacy definition															
Characteristics	5+ medications				10+ medications				15+ medications				20+ medications			
	aPR	99% CI		*p* value	aPR	99% CI		*p* value	aPR	99% CI		*p* value	aPR	99% CI		*p* value
Year	**1.01**	**1.01**	**1.01**	**<0.0001**	**1.03**	**1.02**	**1.03**	**<0.0001**	**1.04**	**1.04**	**1.05**	**<0.0001**	**1.06**	**1.05**	**1.06**	**<0.0001**
Age																
66-75	1	-	-	-	**1**	-	-	**-**	1	-	-	-	1	-	-	**-**
76-85	**1.03**	**1.01**	**1.04**	**<0.0001**	**1.03**	**1.01**	**1.06**	**0.0009**	1.02	0.98	1.07	0.1702	0.98	0.90	1.06	0.4985
86+	**1.03**	**1.01**	**1.05**	**<0.0001**	**1.07**	**1.03**	**1.12**	**<0.0001**	1.02	0.95	1.11	0.4517	0.88	0.76	1.03	0.0335
Sex																
Female	**1.05**	**1.04**	**1.07**	**<0.0001**	**1.12**	**1.08**	**1.15**	**<0.0001**	**1.17**	**1.11**	**1.23**	**<0.0001**	**1.22**	**1.11**	**1.33**	**<0.0001**
Male	1	-	-	-	**1**	-	-	**-**	1	-	-	-	1	-	-	**-**
Combined social and material deprivation																
Least deprived	1	-	-	-	1	-	-	-	1	-	-	-	1	-	-	-
Deprived	1.10	0.98	1.02	0.7063	1.01	0.96	1.05	0.7555	1.05	0.96	1.15	0.1562	1.05	0.90	1.24	0.4046
Mostly social deprived	1.00	0.98	1.02	0.7462	1.01	0.96	1.06	0.6198	1.04	0.95	1.13	0.2441	1.09	0.93	1.28	0.1394
Mostly material deprived	1.01	0.99	1.03	0.4503	1.02	0.97	1.06	0.3460	1.09	1.00	1.19	0.0076	1.06	0.90	1.24	0.3587
Least deprived	1.00	0.98	1.02	0.8444	1.01	0.97	1.06	0.4450	1.10	1.01	1.19	0.0036	1.10	0.95	1.29	0.0974
Missing	1.01	0.99	1.03	0.2489	1.04	1.00	1.10	0.0176	**1.16**	**1.06**	**1.28**	**<0.0001**	1.17	0.99	1.38	0.0126
Number of comorbidities^a^																
0-1	1	-	-	-	**1**	**-**	**-**	**-**	**1**	**-**	**-**	**-**	**1**	**-**	**-**	**-**
2	**1.16**	**1.14**	**1.18**	**<0.0001**	**1.36**	**1.31**	**1.42**	**<0.0001**	**1.60**	**1.47**	**1.74**	**<0.0001**	**1.92**	**1.61**	**2.30**	**<0.0001**
3-4	**1.24**	**1.22**	**1.27**	**<0.0001**	**1.74**	**1.67**	**1.81**	**<0.0001**	**2.51**	**2.31**	**2.72**	**<0.0001**	**3.90**	**3.29**	**4.62**	**<0.0001**
5+	**1.30**	**1.27**	**1.32**	**<0.0001**	**2.14**	**2.06**	**2.23**	**<0.0001**	**4.12**	**3.80**	**4.45**	**<0.0001**	**9.28**	**7.86**	**10.95**	**<0.0001**
Residence area																
Urban																
> 1,000,000 inhab	1	-	-	-	**1**	**-**	**-**	**-**	**1**	**-**	**-**	**-**	**1**	**-**	**-**	**-**
≥ 100,000 inhab	**1.04**	**1.02**	**1.05**	**<0.0001**	**1.10**	**1.06**	**1.13**	**<0.0001**	**1.17**	**1.10**	**1.24**	**<0.0001**	**1.19**	**1.08**	**1.33**	**<0.0001**
≥ 10,000 inhab	**1.05**	**1.03**	**1.07**	**<0.0001**	**1.13**	**1.09**	**1.18**	**<0.0001**	**1.23**	**1.15**	**1.32**	**<0.0001**	**1.20**	**1.05**	**1.36**	**0.0003**
Rural																
<10,000 inhab	**1.04**	**1.03**	**1.06**	**<0.0001**	**1.13**	**1.09**	**1.17**	**<0.0001**	**1.24**	**1.16**	**1.32**	**<0.0001**	**1.31**	**1.17**	**1.47**	**<0.0001**
Missing	**1.05**	**1.02**	**1.09**	**<0.0001**	**1.08**	**1.01**	**1.16**	**0.0056**	0.95	0.82	1.10	0.3417	0.89	0.68	1.16	0.2535

Inhab, inhabitants; aPR, Adjusted prevalence ratio.

^a^ Number of physical and psychiatric comorbidities in a 5-years period (the current year and the 4-years before).

Items in bold indicate statistically significant factors.

## Discussion

The main result of this study is that polypharmacy has been increasing steadily over the last few years for older patients with schizophrenia. To the best of our knowledge, this study is the first one that has evaluated polypharmacy and not only psychiatric polypharmacy (e.g., the use of more than one psychotropic medication) in older individuals with schizophrenia. Some studies have evaluated psychotropic medication use and psychiatric polypharmacy in this population. In a study on schizophrenia-spectrum disorder patients, the authors found that, at hospitalization, 28.1% of patients received four or more psychotropic drugs with a mean number of 2.8 (Gaudiano et al., 2018). Those with four or more psychotropic drugs were older (43.0 vs. 38.6 years) and had more medical comorbidities, including metabolic conditions (Gaudiano et al., 2018). Even if the number of non-psychotropic drugs was not estimated in that study, we could hypothesize that global polypharmacy was significantly higher. A study analyzing prescriptions from office-based physicians in the United States to treat schizophrenia patients showed that 29% of them received at least two medications and 18% three or more (Dussias et al., 2010). Moreover, 58% of patients received one or more antipsychotic medications, and the others received a combination of antipsychotics and other psychiatric medications (20% antidepressants, 15% mood stabilizers, 7% anxiolytics, and 6% treatment for extrapyramidal symptoms) (Dussias et al., 2010). In another study evaluating central nervous system (CNS) medication prescriptions trends in patients with schizophrenia-related conditions between 2004 and 2012 (Heald et al., 2017), the authors found an increase in psychotropic polypharmacy over the study period. This rise corresponded to increased body mass index (BMI) and fasting blood glucose (Heald et al., 2017), conditions requiring additional pharmacological treatments. Despite the lack of studies on global pharmacology in older patients with schizophrenia, the cited studies evaluating psychotropic medications indicate that the pharmacological burden on these patients is significant. Psychiatric polypharmacy could indeed increase the burden of medication load leading to an increase in medications used for both mental and somatic conditions.

In the context of the lack of studies evaluating the global pharmacological burden affecting these patients, our study underlines the high prevalence of polypharmacy, with more than a third of patients having claimed at least 15 different medications in the last year under study. This study should be a starting point in the research on older patients with schizophrenia. Indeed, the long-term pharmacological treatment of these patients should be considered globally in a holistic point of view. The pharmacological treatment should thus be re-evaluated when the patient becomes older. Polypharmacy is a well-known risk factor for many adverse outcomes (Davies et al., 2020; Zaninotto et al., 2020; Franchi et al., 2021; Li et al., 2022). The high proportion of individuals exposed to this potential risk should raise concerns and stimulate new studies on this vulnerable population.

In our population, the rise in polypharmacy was mainly driven by non-antipsychotic medications, for which the use rested stable over time. Some classes, such as medications for osteoporosis or gastrointestinal and cardiovascular drugs, showed an increased use over time. These increases are due to the aging population during the study period and the presence of effective medications on the market (i.e., PPIs). Other classes, such as anxiolytics, showed a significant decrease over time, driven by the changes in clinical guidelines as reported also from a recent population-based study in Quebec (Gosselin et al., 2022). The most used medication classes were those for cardiovascular and respiratory comorbidities, such as heart failure, stroke, asthma, and COPD. Chronic somatic diseases are more frequent among schizophrenia patients than in the general older population. In a review including 25,692 schizophrenia patients, the prevalence of metabolic syndrome was estimated at 32.5%, increasing to 51.9% for patients treated with clozapine (Mitchell et al., 2013). Among older patients with schizophrenia, diabetes is highly prevalent (about 25% of patients), especially among women (Annamalai et al., 2017; Huo et al., 2021), with a 2 to 5-fold increased risk than in the general population (Annamalai et al., 2017; Mamakou et al., 2018). Similarly, these patients are at higher risk for hypertension (Meszaros et al., 2011; Mamakou et al., 2018), obesity (Allison et al., 2009; Annamalai et al., 2017), and dyslipidemia (Mamakou et al., 2018). The higher risk of schizophrenia patients for these comorbidities can be explained by the physiopathology of the disease itself and the utilization of psychotropic medications (Mitchell et al., 2013; Abosi et al., 2018; Mamakou et al., 2018). Antipsychotic medications are indeed associated with an important side effect burden, including metabolic side effects (Jeon and Kim, 2017). Antipsychotic side effects are common, and they may easily reach an intensity requiring another pharmacological treatment, such as benztropine for extrapyramidal side effects (Marder and Cannon, 2019), benzodiazepines, propranolol, or mirtazapine for akathisia, (Zink et al., 2010; Marder and Cannon, 2019), metformin or liraglutide for weight control (de Silva et al., 2016; Grigg et al., 2017), aripiprazole or hormone therapy for hyperprolactinemia (Myles et al., 2017), hormonal therapy for sexual dysfunctions (Grigg et al., 2017; Marder and Cannon, 2019), or laxatives for constipation (De Berardis et al., 2018).

We observed that the number of comorbidities increased over time and contributed to the burden of polypharmacy. This was confirmed by the multivariate regression models analyzing the factors associated with different degrees of polypharmacy. In those analyses, no matter the definition of polypharmacy used, multimorbidity was a statistically and clinically significant factor associated with polypharmacy. The American Psychiatric Association (APA) practice guidelines for managing patients with schizophrenia (Keepers et al., 2020) highlight the importance of addressing integrated medical care to prevent and treat comorbidities. They address weight management, smoke cessation, cardiovascular risk factors (metabolic syndrome, hypertension, dyslipidemia, or heart failure), and renal and liver function. Moreover, these guidelines recommend identifying optimal approaches to prevent and treat specific side effects of antipsychotic medications (i.e., weight gain, metabolic syndrome, cardiovascular toxicity) (Areas for Further Research in Individuals With Schizophrenia, 2021), with particular attention to older individuals for their higher risk for side effects of antipsychotic medications, and potential renal and hepatic impairment (Keepers et al., 2020). Nevertheless, the risks and benefits of exposure to many medications (e.g., polypharmacy) to treat comorbid conditions in older patients with schizophrenia are not well defined yet.

Polypharmacy is a real concern in all older individuals because it has been associated with negative health outcomes such as non-adherence (Franchi et al., 2021), drug-drug interactions (Davies et al., 2020), potentially inappropriate medications (Davies et al., 2020), falls (Zaninotto et al., 2020), hospitalizations (Davies et al., 2020), and mortality (Li et al., 2022), also in COVID-19 patients (Sirois et al., 2022). In this schizophrenia patients, the use of antipsychotic medications, which are necessary to control the symptoms of the disease, can increase the risk for physical comorbidities, especially cardiovascular and metabolic ones (Jeon and Kim, 2017). Older patients with schizophrenia represent a real challenge because of the high number of medications they receive for schizophrenia itself and the frequent comorbidities they are diagnosed with. Future studies should identify the effect of polypharmacy on the risk of negative health outcomes and mortality. They should also focus on which combinations of medications can provide the greatest benefits with the lowest risks for better integrated medical care, considering not only the control of schizophrenia and the management of its treatment with antipsychotics and psychiatric medications but also non-psychiatric comorbidities, their prevention and treatment.

This study highlighted how polypharmacy is frequent in older adults with schizophrenia, even when more restrictive thresholds as 15 or 20 medications and above are used to define it. These patients are indeed at elevated risk for drug-drug interactions, adverse drug effects interactions, and drug-disease interactions compared to their peers without schizophrenia because of the frequency of physical comorbidities and the already impacting burden of antipsychotic treatments.

We believe this study has the main strength of well highlighting the burden of global polypharmacy among older individuals with schizophrenia. Medico-administrative databases allowed us to access annual large cohorts of patients with schizophrenia throughout Québec, as well as all the reimbursed medications they claimed, the diagnoses they received, and their resource utilization. With this approach, we could observe the pharmacological burden of older patients with schizophrenia, putting the antipsychotic treatment in the context of the global treatment of the older individual. Moreover, we could analyze trends and patterns of different pharmacological classes over a period of almost 20 years, highlighting changes and practices.

The results of this study should, nevertheless, be considered in light of some limitations. First, because of the use of administrative databases, we could not clinically assess the diagnosis of schizophrenia or the presence of comorbidities. However, the algorithms used for the identification of such diagnoses are routinely used by the INSPQ for surveillance purposes and the QICDSS (Blais et al., 2014). We could also have overestimated polypharmacy. To measure polypharmacy, we used claims of prescribed medications during a 1-year time frame. This means that the medications considered could not have been used simultaneously, as happens when treatments are switched because of side effects or inefficacy. On the contrary, we could consider only prescribed medications reimbursed by the public drug plan. This could have thus led to an underestimation of polypharmacy since over-the-counter medications, such as anti-inflammatory drugs, or laxatives, have not been considered among the medications accounting for polypharmacy. Still, since the same operational definition of polypharmacy was used for every year of the study, the conclusion on the increasing burden of medications among older individuals with schizophrenia persist, with the same overestimation of individuals exposed to polypharmacy being homogenous over time. Finally, this study aimed to explore global polypharmacy in older patients with schizophrenia, its prevalence, trends, and patterns over time, and it was thus designed for these purposes only. Therefore, we did not assess the effects of polypharmacy, such as adverse events, hospitalizations or mortality in this population.

## Conclusion

We found a noticeable increase in polypharmacy exposure in older adults with schizophrenia, with the proportion of subjects having claimed at least 5, 10, 15, and 20 medications increasing to about 90%, 60%, 35%, and 15% in 2016. This raises concerns about the growing risks of adverse effects and drug-drug interactions that could arise in these patients, especially considering the use of antipsychotic treatments.

The risks and benefits of polypharmacy in older patients with schizophrenia are not well defined yet. There is a need to better understand which combinations of medications provide the greatest benefits and lowest risks and consider the presence of non-psychiatric comorbidities and the concomitant use of psychiatric and non-psychiatric drugs.

## Data Availability

The data analyzed in this study is subject to the following licenses/restrictions: We do not have permission to share the data from the Quebec Integrated Chronic Diseases Surveillance System (QICDSS). Requests to access these datasets should be directed to the Quebec Information access commissioner—Commission d’accès à l’information du Québec.

## Supplementary material

SUPPLEMENTAL FIGURE S1 Graphical representation of the combined deprivation index. The combined deprivation index combines social and material deprivation into a grouped deprivation index. Green: most deprived; Yellow: deprived; Blue: mostly socially deprived Orange: mostly materially deprived; Purple: least deprived.
