# Supplementary figures and images for: Psychiatric and non-psychiatric polypharmacy among older adults with schizophrenia: Trends from a population-based study between 2000 and 2016
Source: Front Pharmacol. 2023 Feb 7;14:1080073. doi: 10.3389/fphar.2023.1080073 (PMC9941679; doi:10.3389/fphar.2023.1080073)

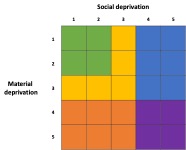

Supplement: Supplementary file 1 [file Image1.JPEG]
